# TIG welding of Ti6Al4V alloy: Microstructure, fractography, tensile and microhardness data

**DOI:** 10.1016/j.dib.2021.107274

**Published:** 2021-08-11

**Authors:** Peter Omoniyi, Modupe Mahamood, Tien-Chien Jen, Esther Akinlabi

**Affiliations:** a Department of Mechanical Engineering Science, University of Johannesburg, South Africa; b Department of Mechanical Engineering, University of Ilorin, Nigeria; c Department of Materials and Metallurgical Engineering, University of Ilorin, Nigeria; d Pan African University for Life and Earth sciences Institute (PAULESI), Ibadan, Nigeria

**Keywords:** Fractography, Microhardness, Microstructure, Tensile Strength, Ti6Al4V, TIG

## Abstract

Titanium alloy is widely used in many industries due to its unique weight to strength ratio and high corrosion resistance. A suitable method of joining Titanium and its alloys is using Tungsten Inert Gas (TIG) welding. A significant advantage of TIG welding over other fusion welding is its ability to use non-consumable electrodes. This research was carried out on Ti6Al4V alloy of 2-3 mm thickness using TIG welding. Current and gas flow rates were varied, with Argon used as the inert gas for the welding. The data and images presented in this article account for the mechanical (tensile and microhardness), fractography, and microstructural properties of the welded samples. This article will foster simulation of heat input and flux and mechanical properties of TIG-welded Ti6Al4V alloy.

**Specifications Table**

**Table:** 

Subject	Mechanical Engineering, Industrial Engineering, Material Science
Specific subject area	Manufacturing Engineering, Welding and Fabrication
Type of data	Table Chart Image Graph Figure
How data were acquired	Tensile data were acquired from the Universal Testing Machine (UTM) Zwick Roell 2250. Microhardness data acquired from the Indentec Digital Vickers microhardness tester. Microstructure images were captured using Olympus DP25 Optical Microscope, and the fractography images were obtained from the TESCAN SEM machine. Macrostructure images were acquired using the Olympus SZX16 macroscope, and the bead geometry was measured using the Olympus Stream Essentials software.
Data format	Analyzed Raw
Parameters for data collection	The current used for Ti6Al4V alloy of 2 mm thickness was varied between 50-60 A, and gas flowrate varied between 7-9 L/min. For 3 mm thickness, current: 100-120 A and gas flow rate between 9-12 L/min. For both thicknesses, the gas used in welding was Argon with 99.99% purity, Thorium electrode, and Ti6Al4V ELI alloy as filler metal.
Description of data collection	Microhardness was carried out using Indentec digital Vickers microhardness tester. A force of 4.9 N and a dwell time of 15 s was used. Tensile strength was determined using UTM Zwick Roell 2250, and the test was done according to the ASTM E8 standard.
Data source location	Institution: University of Johannesburg City/Town/Region: Johannesburg/Gauteng Country: South Africa Latitude and longitude (and GPS coordinates, if possible) for collected samples/data: S26 10 54.9 E27 59 53.9
Data accessibility	With the article

## Value of the Data

• In conjunction with the images, the dataset will further give an insight into the simulation and analysis of the strength of the material.
• Industries such as automobile, chemical, marine, and aerospace will benefit from the data supplied since the primary material used by these industries is Titanium due to its unique properties over other metals.
• The dataset and images can give the welders/engineers vital information in various industries where Titanium alloy is used.
• The experiment was carefully selected and designed using the Taguchi design of experiment method. Hence, It will guide the engineers/welders on designing, planning, and executing welding operations.
• The data can aid in the development an empirical model for predicting and optimizing TIG welding of Ti6Al4V alloy.

## 1 Data Description

**Fig. 1:**
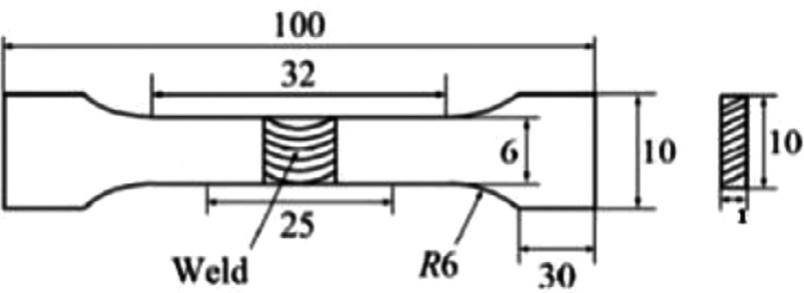
Tensile sample (ASTM E8 sub-size).

**Fig. 2:**
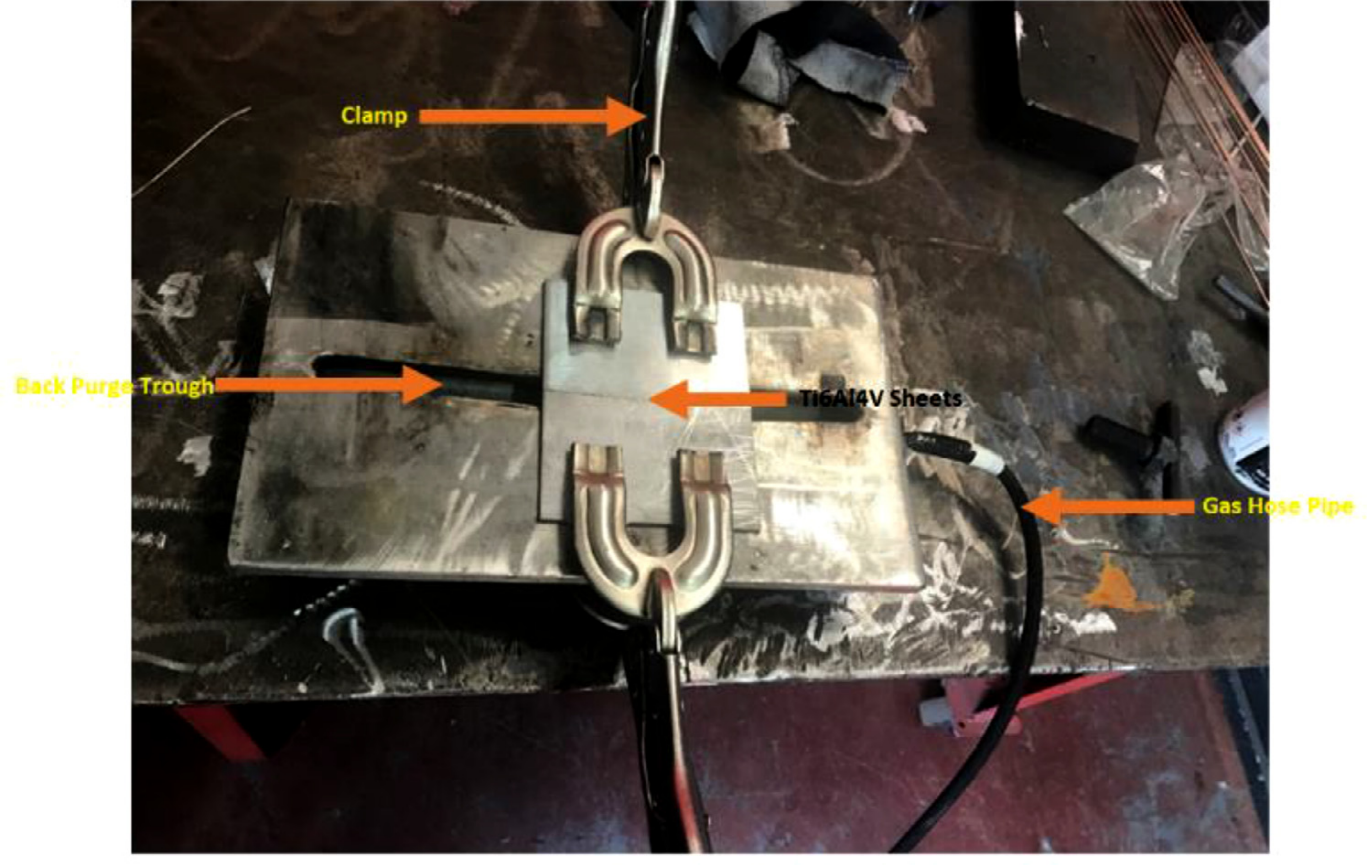
Experimental setup.

A commercial mill annealed titanium grade 5 alloy sheet measuring 100 × 60 × 3 mm and 100 × 60 × 2 mm were the material used in this experiment. Tables 1 and 2 show the chemical composition of the Ti6Al4V sheets and the Ti6Al4V ELI filler rod used for this experiment, which was obtained from the material safety data sheet from the supplier. The materials conforms with ASTM B265 [1] and ASTM F136 [2], respectively. Table 3 presents the mechanical properties of the Ti6Al4V sheet and Ti6Al4V ELI filler rods used at room temperature. Table 4, Table 5 show the experimental design for welding 3 mm and 2 mm thick sheets, respectively. The experimental design was adopted based on the optimum range of parameters recommended by Muncaster [3], and the parameters were randomized using the Taguchi L4 (2^2^) design of experiment. The ASTM E8 [4] sub-size was used for the tensile test, as shown in Figure 1. There is a provision for gas flow beneath the welded plate to prevent back purging and contamination of weld, as shown in the experimental setup Figure 2.

**Table 1:** Chemical composition of Ti6Al4V alloy sheet.

Element	Ti	Al	V	Fe	C	N	H	O	Others
Weight (%)	Remainder	6.10	4.0	0.15	0.03	0.018	0.002	0.13	Each<0.10

**Table 2:** Chemical composition of Ti6Al4V ELI filler rod.

Element	Ti	Al	V	Fe	C	N	H	O	Others
Weight (%)	Remainder	6.14	4.15	0.125	0.015	0.003	0.001	0.10	Each<0.30

**Table 3:** Mechanical properties of Ti6Al4V sheet alloy and Ti6Al4V ELI filler rod.

Alloy	Parameters	Tensile Strength (MPa)	Yield Strength (MPa)	Elongation (%)	Microhardness (HV)
Ti6Al4V	Values	895	825	10	362
Ti6Al4V ELI		860	795	17	341

**Table 4:** Experimental Process Parameters for TIG welding of 3 mm thick Ti6Al4V sheets (Category A).

Sample No.	Current (A)	Gas Flow Rate (L/min)
T31	100	9
T32	120	9
T33	120	12
T34	100	12

**Table 5:** Experimental Process Parameters for TIG welding of 2 mm thick Ti6Al4V sheets (Category B).

Sample No.	Current (A)	Gas Flow Rate (L/min)
T21	60	9
T22	50	9
T23	50	7
T24	60	7

**Fig. 3:**
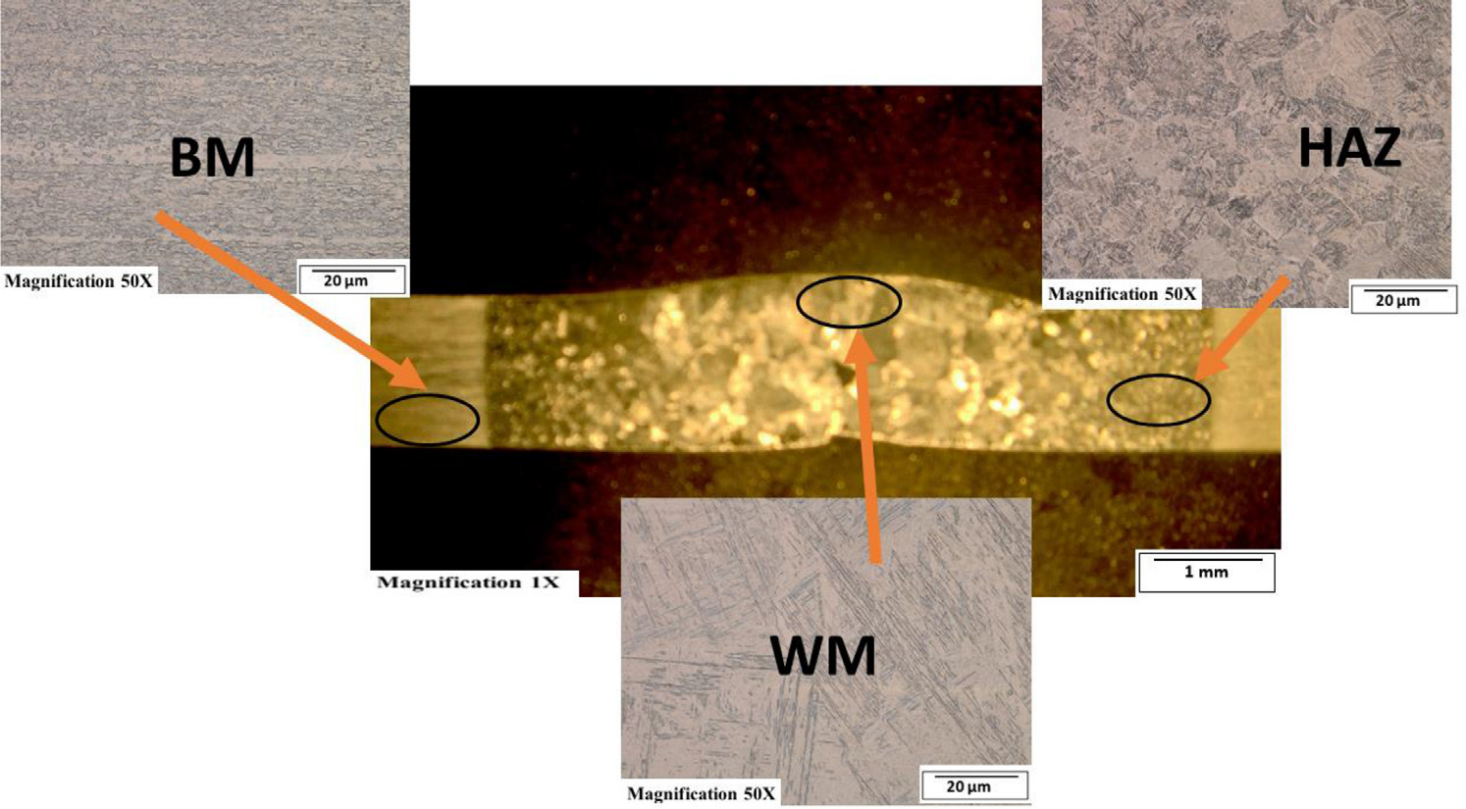
Micrograph of sample T34.

**Fig. 4:**
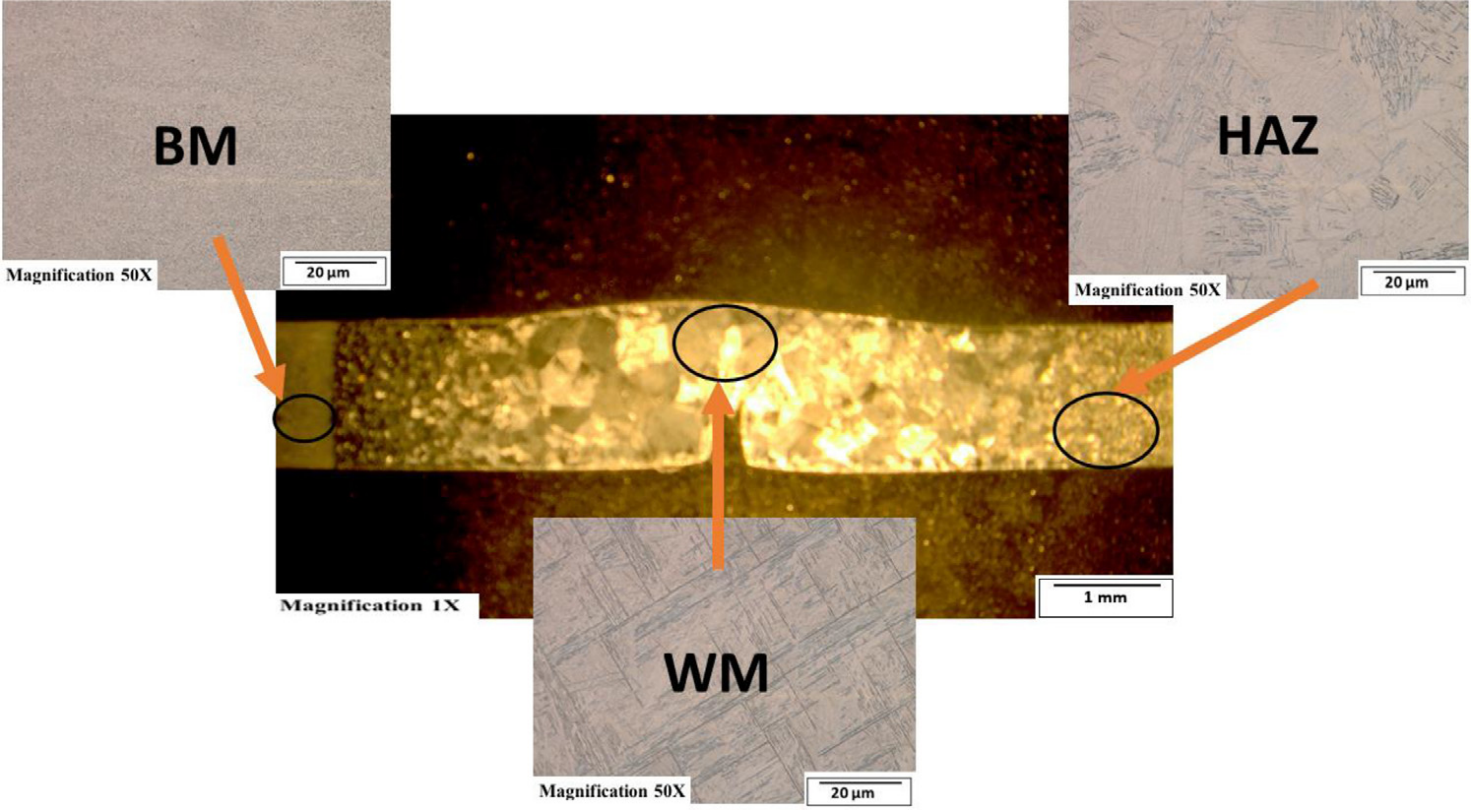
Micrograph of sample T33.

**Fig. 5:**
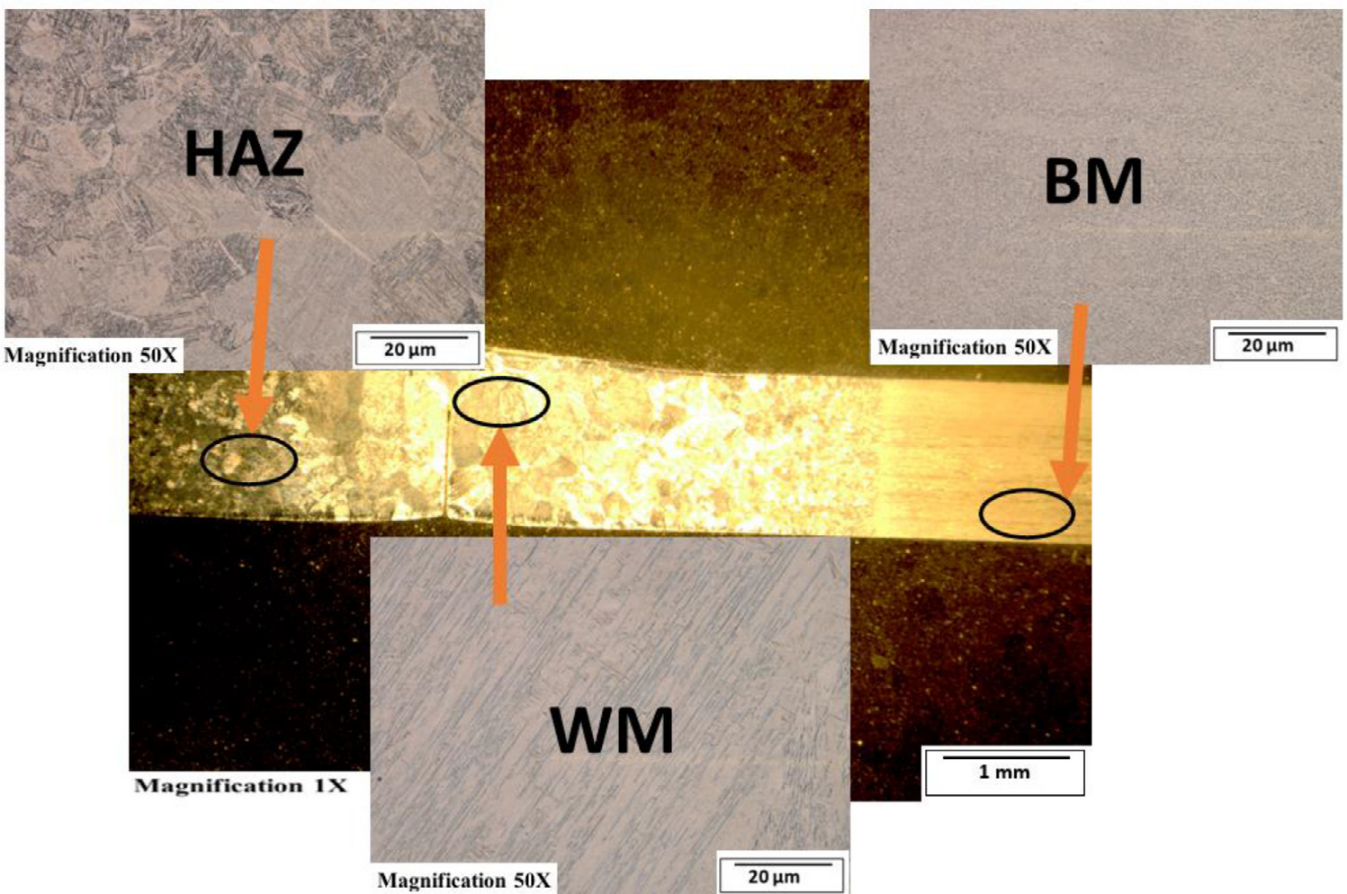
Micrograph of sample T32.

**Fig. 6:**
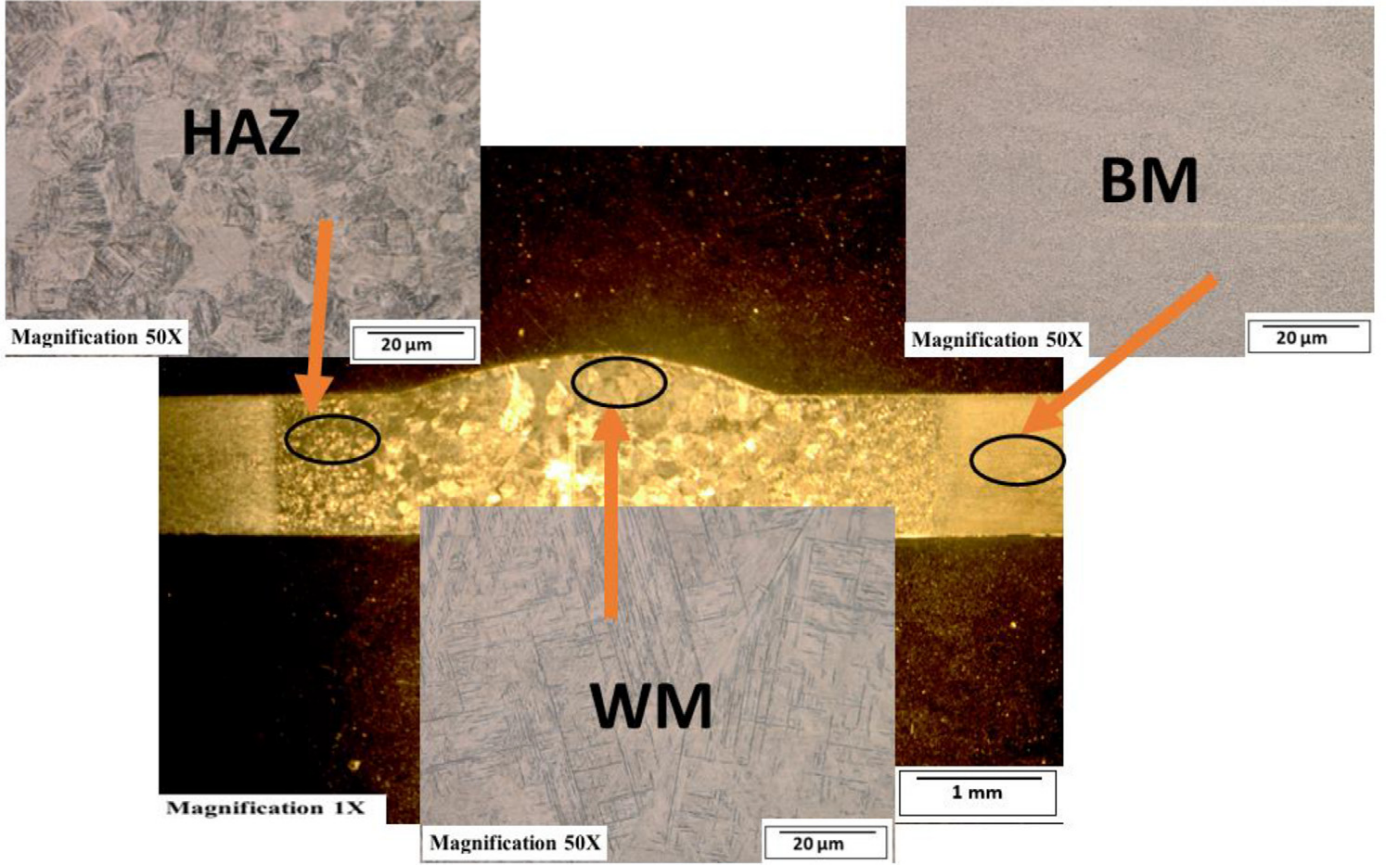
Micrograph of sample T31.

**Fig. 7:**
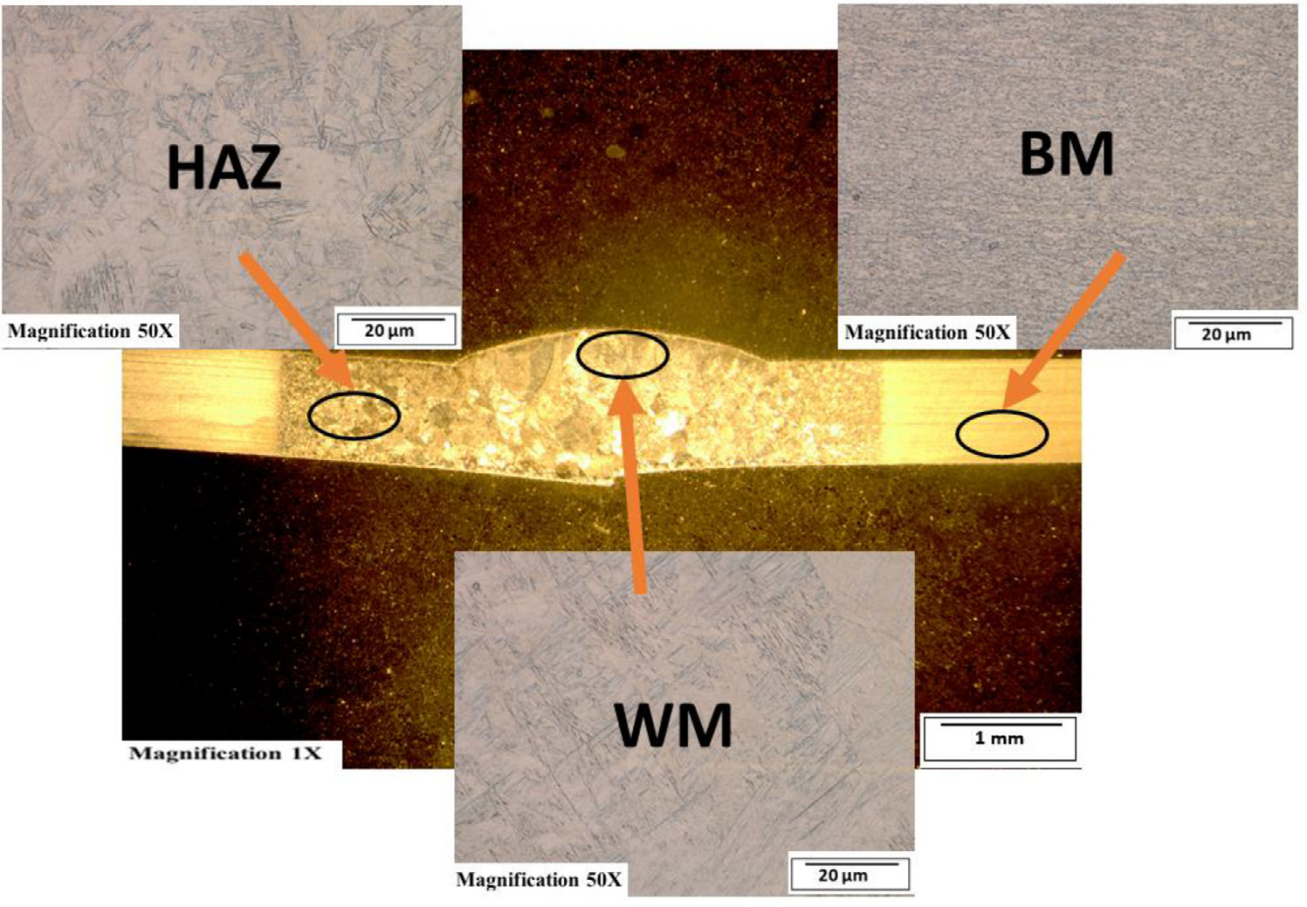
Micrograph of sample T24.

**Fig. 8:**
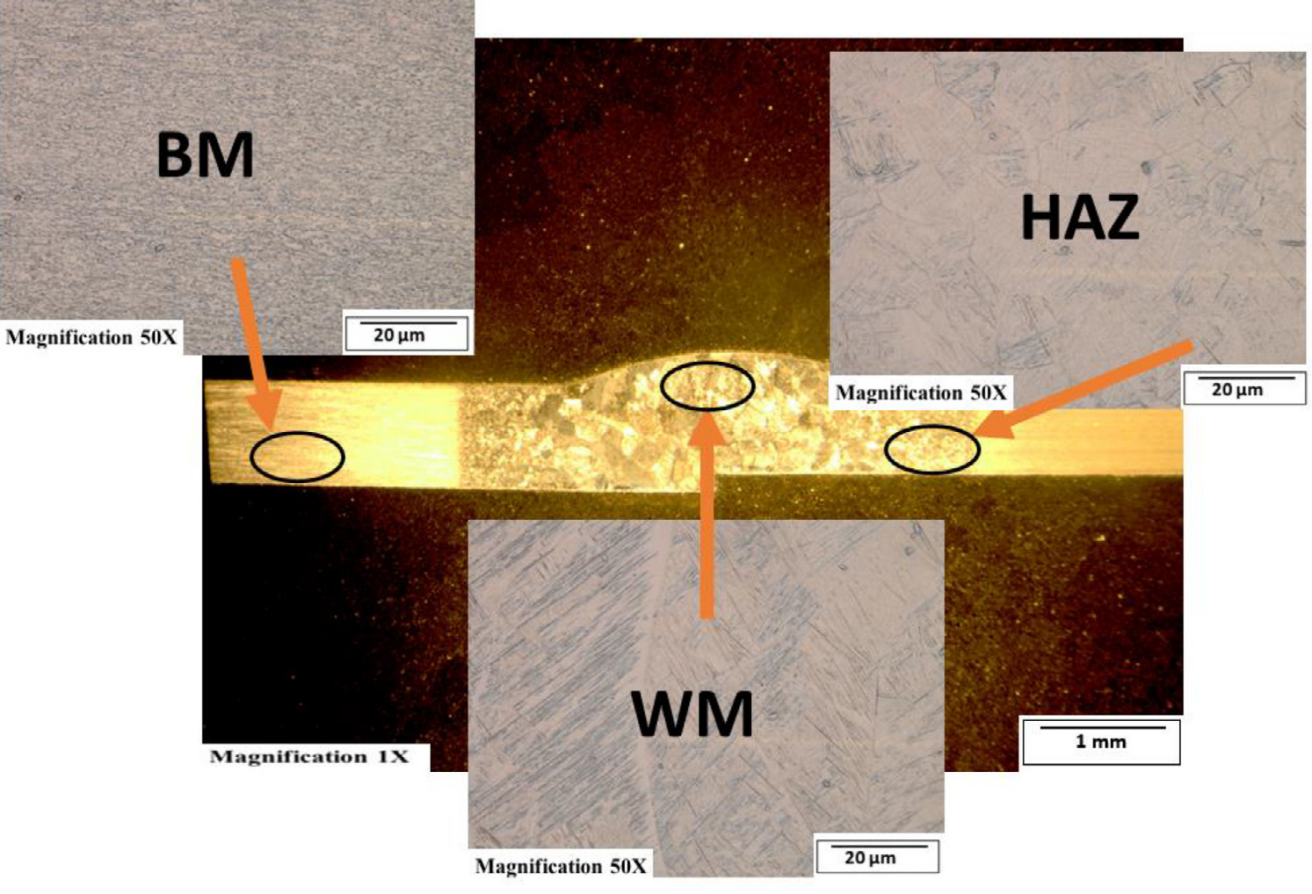
Micrograph of sample T23.

**Fig. 9:**
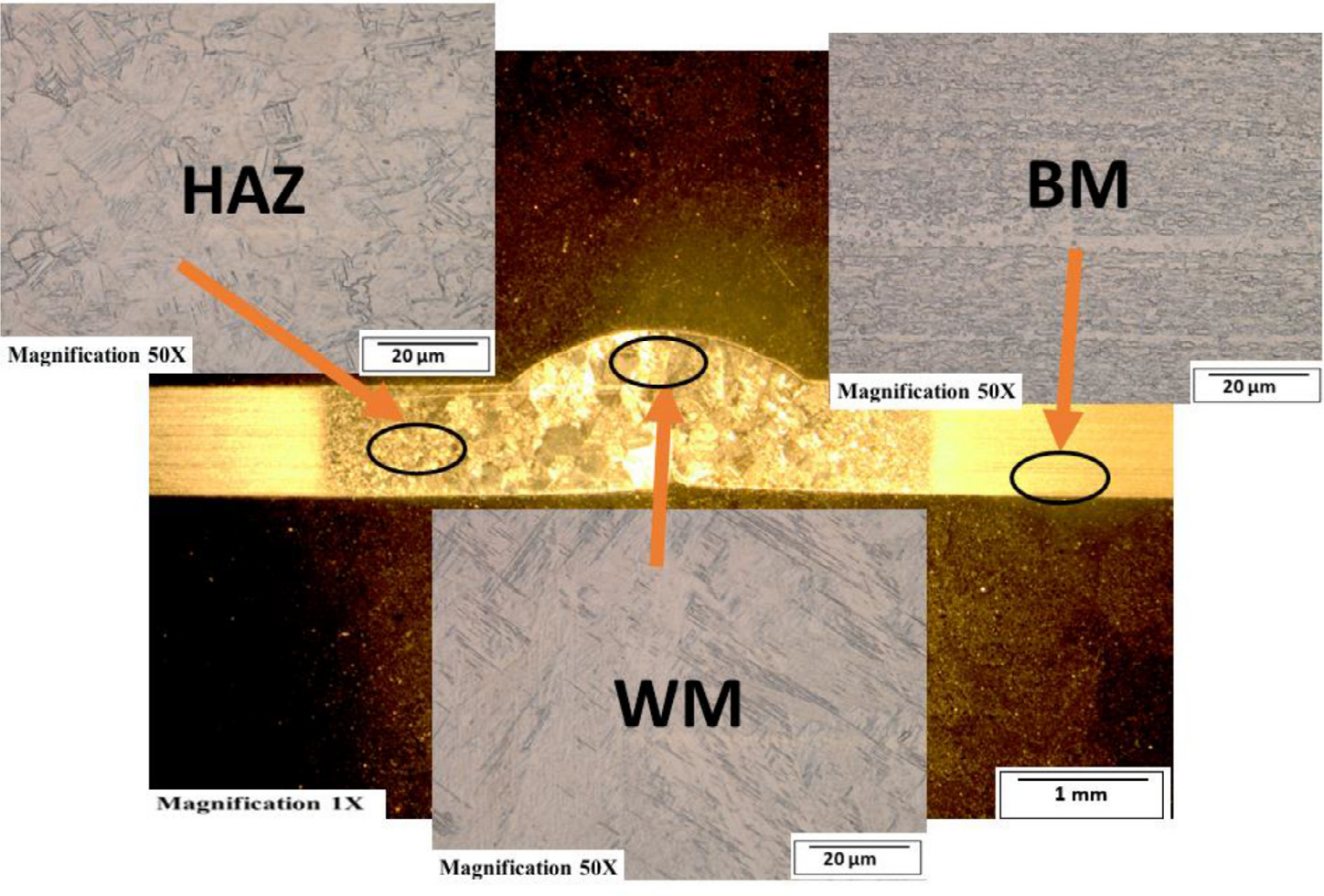
Micrograph of sample T22.

**Fig. 10:**
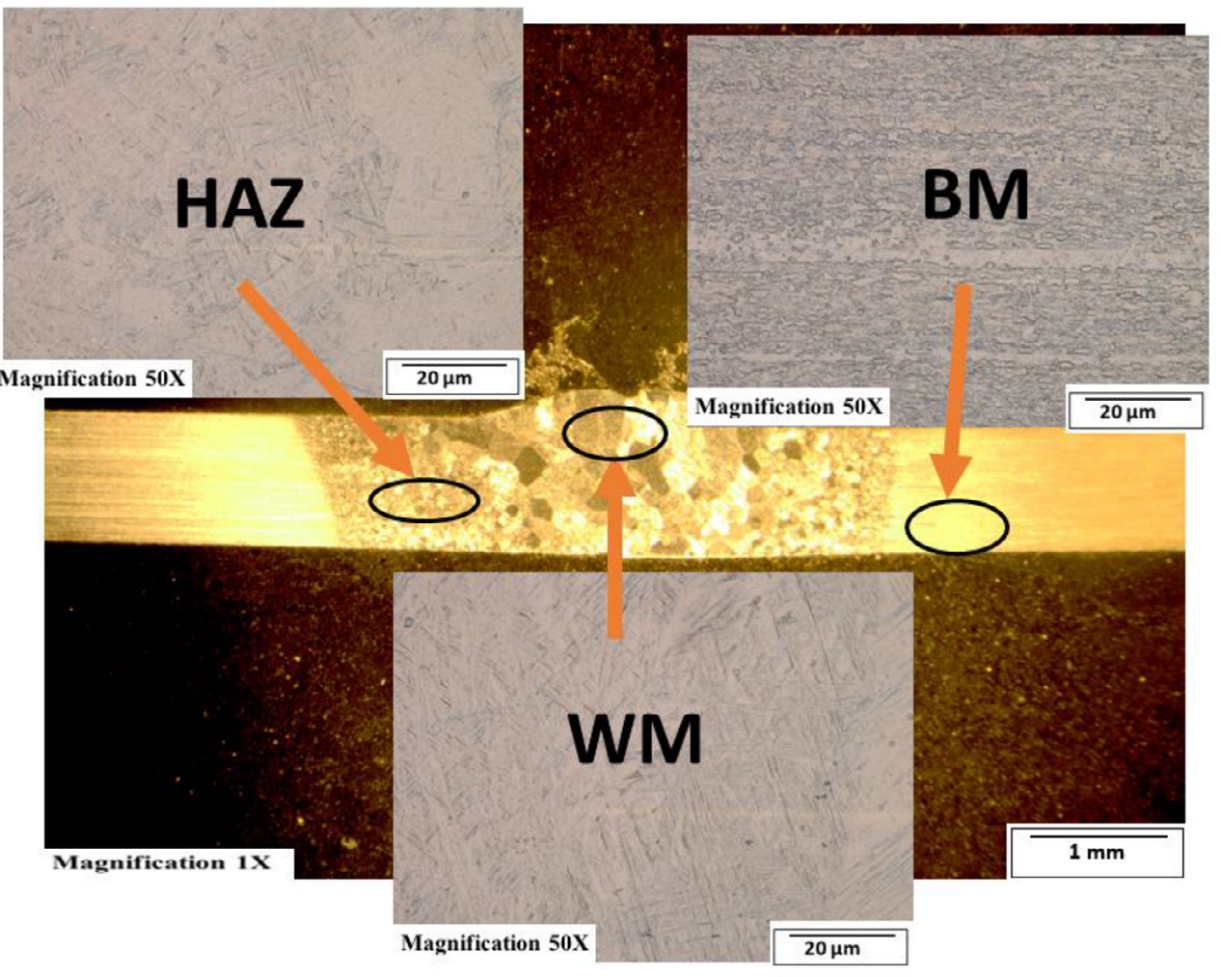
Micrograph of sample T21.

**Fig. 11:**
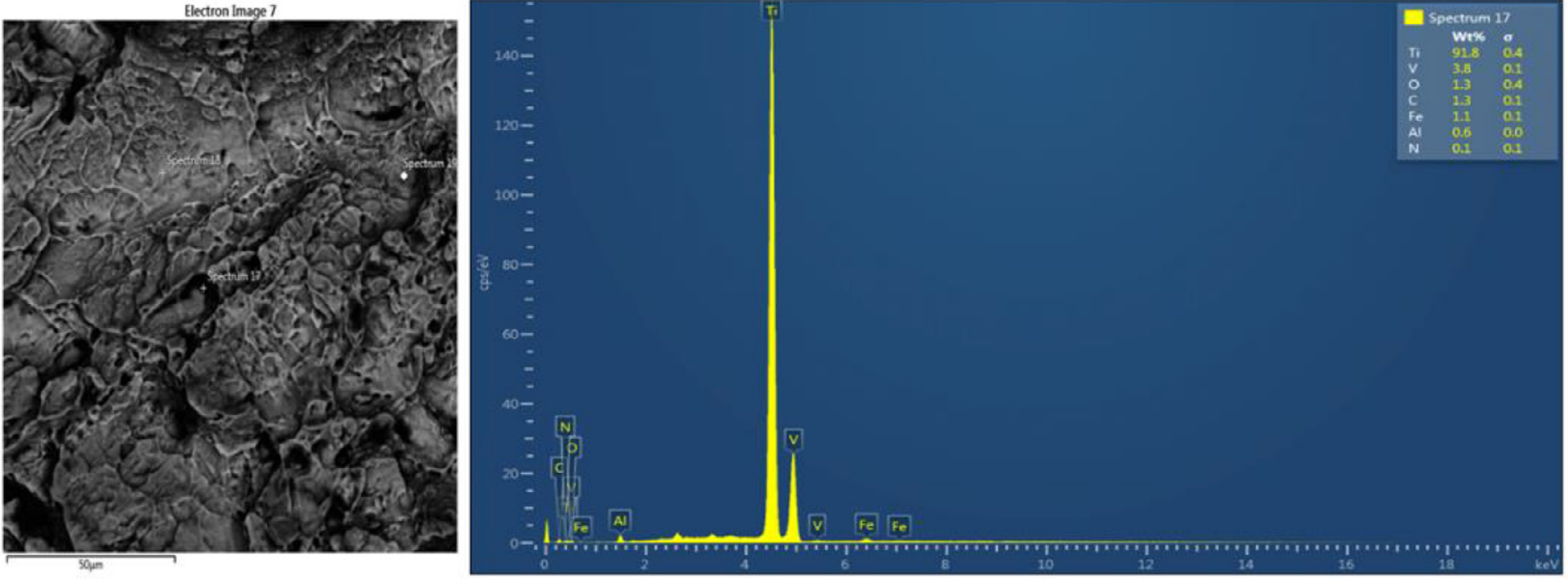
Fractograph of 2 mm thick Ti6Al4V alloy fractured at FZ.

**Fig. 12:**
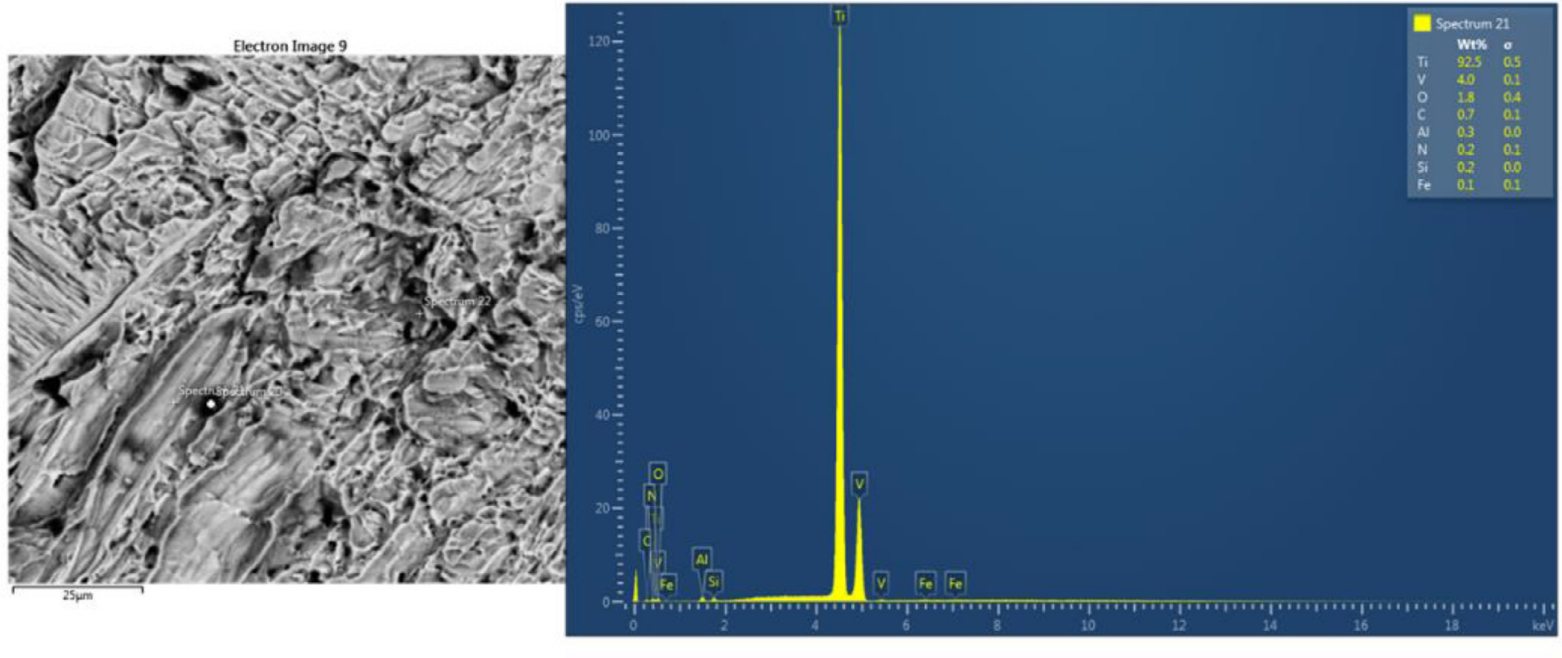
Fractograph of 3 mm thick Ti6Al4V alloy fractured at FZ.

**Fig. 13:**
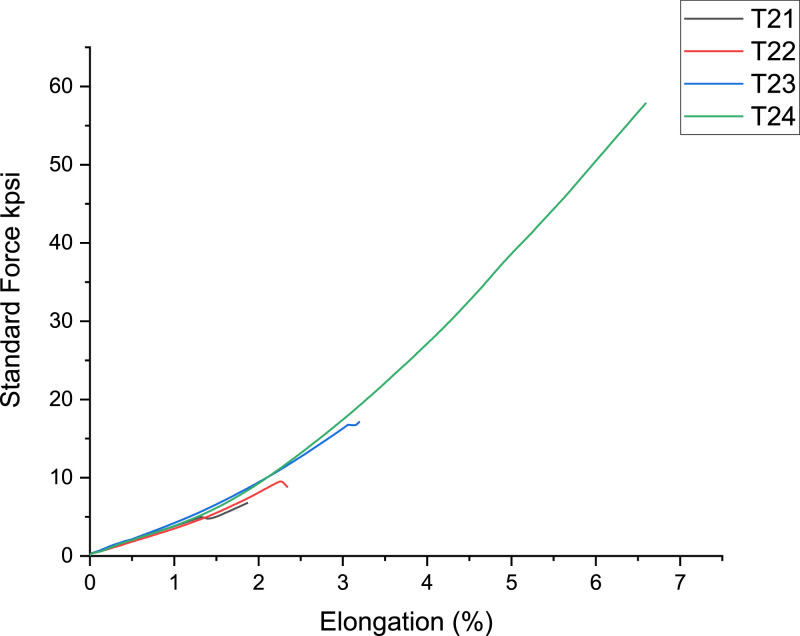
Stress-strain curve for 2 mm thick Ti6Al4V alloy.

**Fig. 14:**
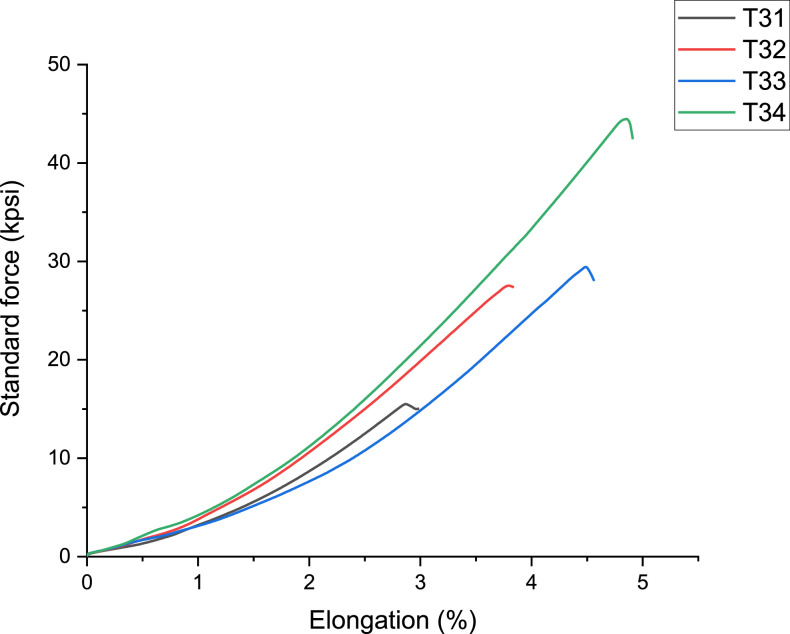
Stress-strain curve for 3 mm thick Ti6Al4V alloy.

**Fig. 15:**
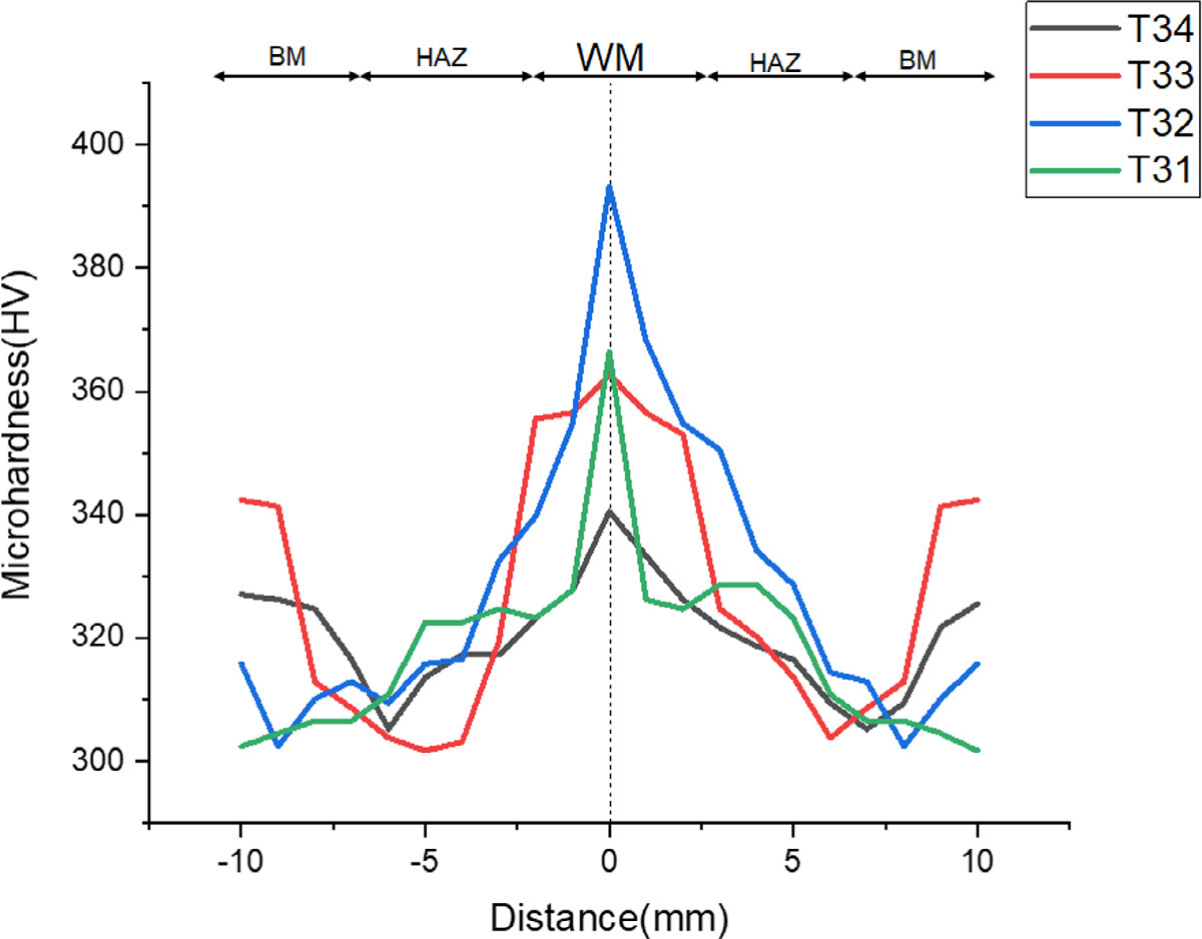
Microhardness profile of 3 mm thick Ti6Al4V alloy.

**Fig. 16:**
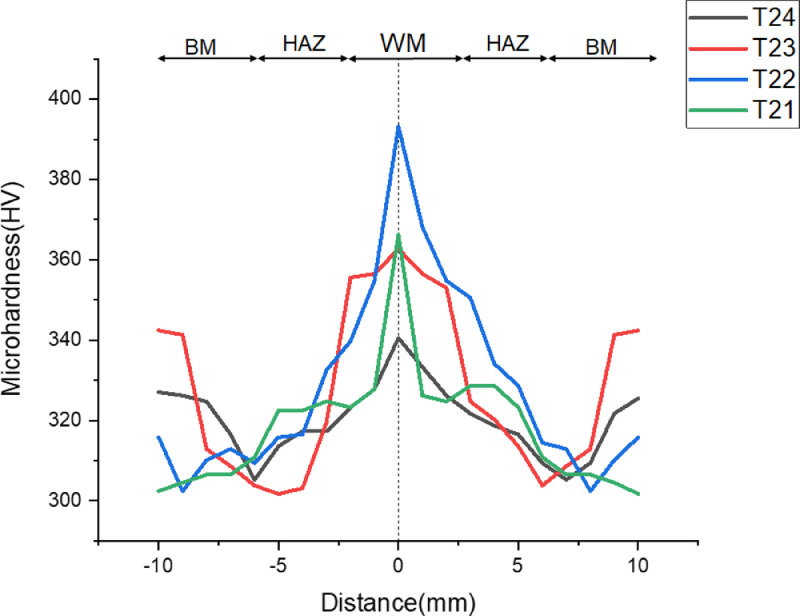
Microhardness profile of 2 mm thick Ti6Al4V alloy.

Figure 3-10 shows the images of the microstructure of each sample. Each image shows the microstructure at three different zones, namely, heat affected zone (HAZ), base metal (BM), and fusion zone (FZ). The bead geometry measured using the Olympus Stream Essentials software is shown in Table 6, and the fractography Images of the failed samples at FZ are shown in Figures 11 and 12 for 2 and 3 mm, respectively. The tensile and raw microhardness values are presented in the supplementary files. Furthermore, Figures 13 and 14 shows the stress-strain relationship for 2 and 3 mm thick alloy, derived from the raw data in the supplementary file. Lastly, Figures 15 and 16 present the microhardness profile derived from the raw data presented in the supplementary file for 2 and 3 mm thick alloy, respectively.

**Table 6:** Bead geometry of TIG-welded samples.

Sample	Bead width (µm)	Bead height (µm)
T34	3665.20	1683.00
T33	4192.20	1132.20
T32	4539.00	605.20
T31	3682.20	714.00
T24	2890.00	1366.80
T23	2485.40	1071.00
T22	2550.00	1203.60
T21	2451.40	1275.00

## 2 Experimental Design, Materials and Methods

The TIG welding done in this experiment was carried out using the Afrox industrial 175 multiprocess welding machine and were done in two categories, Ti6Al4V sheets of 100 × 60 × 3 mm were joined as category A and Ti6Al4V sheets of 100 × 60 × 2 mm as category B. The two categories were joined using Ti6Al4V ELI as filler metal. Pure Argon of 99.99% purity was used as the inert gas to prevent the weld's back purging and oxygen contamination [5]. Before welding, each sheet was cleaned with acetone to remove oxide films and contaminations from its surface [6]. Four welds were done, varying current and gas flow rate for each category.

Each sample's microstructural examination was done using a cut out of 25 × 10 mm for each thickness. Each sample was ground using Silicon Carbide (SiC) papers of different sizes (#320-500-800-1200). They were then polished and etched using Kroll's reagent containing 85% H_2_O + 10% HNO_3_ + 5% HF for 18s [7,8]. The images were captured at 50X magnification at the heat affected zone (HAZ), base metal (BM), and the fusion zone (FZ) for each thickness. Furthermore, macrostructure images were taken at 1X to view the weld zone (WZ) and measure the bead geometry using the Olympus Stream Essentials software.

A Microhardness test was conducted at different WZ to create a hardness profile. Twenty indentations were done at a 1mm interval from each other, with a force of 4.9 N and a dwell time of 15s, following ASTM E384 [9].

Tensile tests were carried out using the UTM Zwick Roell 2250, and following ASTM E8 [4,10,11], three samples were used for this test for each welding parameter. The samples were further analyzed at the failure point by obtaining the fractography images using the TESCAN SEM to study the manner of failure of the material.

## Ethics Statement

The research was not performed on humans, animals or conducted using social media.

## CRediT Author Statement

**Peter Omoniyi:** Writing-original draft preparation, Methodology, Data curation, Investigation, Software; **Modupe Mahamood:** Editing, Writing-Reviewing and Supervision; **Tien-Chien Jen:** Supervision; **Esther Akinlabi:** Supervision, Conceptualization, Methodology, Writing-Reviewing, and Editing.

## Declaration of Competing Interest

The authors state that there are no known competing financial interests or personal ties that may have influenced the work described in this article.

## Appendix Supplementary materials

Image, application 1

Image, application 2
